# On the k-synchronizability of Systems

**DOI:** 10.1007/978-3-030-45231-5_9

**Published:** 2020-04-17

**Authors:** Cinzia Di Giusto, Laetitia Laversa, Etienne Lozes

**Affiliations:** 8 ENS/CNRS, Université Paris-Saclay, Cachan, France; 9 University of Duisburg-Essen, Duisburg, Germany; Université Côte d’Azur, CNRS, I3S, Sophia Antipolis, France

**Keywords:** Verification, Communicating Automata, A/Synchronous communication

## Abstract

We study *k*-synchronizability: a system is *k*-synchronizable if any of its executions, up to reordering causally independent actions, can be divided into a succession of *k*-bounded interaction phases. We show two results (both for mailbox and peer-to-peer automata): first, the reachability problem is decidable for *k*-synchronizable systems; second, the membership problem (whether a given system is *k*-synchronizable) is decidable as well. Our proofs fix several important issues in previous attempts to prove these two results for mailbox automata.

## 1 Introduction

Asynchronous message-passing is ubiquitous in communication-centric systems; these include high-performance computing, distributed memory management, event-driven programming, or web services orchestration. One of the parameters that play an important role in these systems is whether the number of pending sent messages can be bounded in a predictable fashion, or whether the buffering capacity offered by the communication layer should be unlimited. Clearly, when considering implementation, testing, or verification, bounded asynchrony is preferred over unbounded asynchrony. Indeed, for bounded systems, reachability analysis and invariants inference can be solved by regular model-checking [5]. Unfortunately and even if designing a new system in this setting is easier, this is not the case when considering that the buffering capacity is unbounded, or that the bound is not known a priori . Thus, a question that arises naturally is how can we bound the "behaviour" of a system so that it operates as one with unbounded buffers? In a recent work [4], Bouajjani *et al.* introduced the notion of $k$-synchronizable system of finite state machines communicating through mailboxes and showed that the reachability problem is decidable for such systems. Intuitively, a system is $k$-synchronizable if any of its executions, up to reordering causally independent actions, can be chopped into a succession of $k$-bounded interaction phases. Each of these phases starts with at most $k$ send actions that are followed by at most $k$ receptions. Notice that, a system may be $k$-synchronizable even if some of its executions require buffers of unbounded capacity.

As explained in the present paper, this result, although valid, is surprisingly non-trivial, mostly due to complications introduced by the mailbox semantics of communications. Some of these complications were missed by Bouajjani *et al.* and the algorithm for the reachability problem in [4] suffers from false positives. Another problem is the membership problem for the subclass of $k$-synchronizable systems: for a given $k$ and a given system of communicating finite state machines, is this system $k$-synchronizable? The main result in [4] is that this problem is decidable. However, again, the proof of this result contains an important flaw at the very first step that breaks all subsequent developments; as a consequence, the algorithm given in [4] produces both false positives and false negatives.

In this work, we present a new proof of the decidability of the reachability problem together with a new proof of the decidability of the membership problem. Quite surprisingly, the reachability problem is more demanding in terms of causality analysis, whereas the membership problem, although rather intricate, builds on a simpler dependency analysis. We also extend both decidability results to the case of peer-to-peer communication.

**Outline.** Next section recalls the definition of communicating systems and related notions. In Section 3 we introduce $k$-synchronizability and we give a graphical characterisation of this property. This characterisation corrects Theorem 1 in [4] and highlights the flaw in the proof of the membership problem. Next, in Section 4, we establish the decidability of the reachability problem, which is the core of our contribution and departs considerably from [4]. In Section 5, we show the decidability of the membership problem. Section 6 extends previous results to the peer-to-peer setting. Finally Section 7 concludes the paper discussing other related works. Proofs and some additional material are available at https://hal.archives-ouvertes.fr/hal-02272347.

## 2 Preliminaries

A communicating system is a set of finite state machines that exchange messages: automata have transitions labelled with either send or receive actions. The paper mainly considers as communication architecture, mailboxes: i.e., messages await to be received in FIFO buffers that store all messages sent to a same automaton, regardless of their senders. Section 6, instead, treats peer-to-peer systems, their introduction is therefore delayed to that point.

Let $\mathbb{V}$ be a finite set of messages and $\mathbb{P}$ a finite set of processes. A send action, denoted $send(p, q, \mathbf{v})$, designates the sending of message $\mathbf{v}$ from process $p$ to process $q$. Similarly a receive action $rec(p, q, \mathbf{v})$ expresses that process $q$ is receiving message $\mathbf{v}$ from $p$. We write $a$ to denote a send or receive action. Let $S = \{send(p, q, \mathbf{v}) \mid p, q \in \mathbb{P}, \mathbf{v} \in \mathbb{V}\}$ be the set of send actions and $R = \{rec(p, q, \mathbf{v}) \mid p, q \in \mathbb{P}, \mathbf{v} \in \mathbb{V}\}$ the set of receive actions. $S_p$ and $R_p$ stand for the set of sends and receives of process $p$ respectively. Each process is encoded by an automaton and by abuse of notation we say that a *system* is the parallel composition of processes.

**Definition 1 (System).** *A system is a tuple* $\mathfrak{S} = \big((L_p, \delta_p, l_p^0) \mid p \in \mathbb{P}\big)$ *where, for each process* $p$*,* $L_p$ *is a finite set of local control states,* $\delta_p \subseteq (L_p \times (S_p \cup R_p) \times L_p)$ *is the transition relation (also denoted* $l \xrightarrow{a}_p l'$*) and* $l_p^0$ *is the initial state.*

**Definition 2 (Configuration).** *Let* $\mathfrak{S} = \big((L_p, \delta_p, l_p^0) \mid p \in \mathbb{P}\big)$*, a configuration is a pair* $(\vec{l}, \texttt{Buf})$ *where* $\vec{l} = (l_p)_{p\in\mathbb{P}} \in \Pi_{p\in\mathbb{P}} L_p$ *is a global control state of* $\mathfrak{S}$ *(a local control state for each automaton), and* $\texttt{Buf} = (b_p)_{p\in\mathbb{P}} \in (\mathbb{V}^*)^{\mathbb{P}}$ *is a vector of buffers, each* $b_p$ *being a word over* $\mathbb{V}$*.*

We write $\vec{l_0}$ to denote the vector of initial states of all processes $p \in \mathbb{P}$, and $\texttt{Buf}_0$ stands for the vector of empty buffers. The semantics of a system is defined by the two rules below.

[SEND]

$$\frac{l_p \xrightarrow{send(p,q,\mathbf{v})}_p l'_p \quad b'_q = b_q \cdot \mathbf{v}}{(\vec{l}, \texttt{Buf}) \xrightarrow{send(p,q,\mathbf{v})} (\vec{l}[l'_p/l_p], \texttt{Buf}[b'_q/b_q])}$$

[RECEIVE]

$$\frac{l_q \xrightarrow{rec(p,q,\mathbf{v})}_q l'_q \quad b_q = \mathbf{v} \cdot b'_q}{(\vec{l}, \texttt{Buf}) \xrightarrow{rec(p,q,\mathbf{v})} (\vec{l}[l'_q/l_q], \texttt{Buf}[b'_q/b_q])}$$

A send action adds a message in the buffer $b$ of the receiver, and a receive action pops the message from this buffer. An execution $e = a_1 \cdots a_n$ is a sequence of actions in $S \cup R$ such that $(\vec{l_0}, \texttt{Buf}_0) \xrightarrow{a_1} \cdots \xrightarrow{a_n} (\vec{l}, \texttt{Buf})$ for some $\vec{l}$ and $\texttt{Buf}$. As usual $\stackrel{e}{\Rightarrow}$ stands for $\xrightarrow{a_1} \cdots \xrightarrow{a_n}$. We write $asEx(\mathfrak{S})$ to denote the set of asynchronous executions of a system $\mathfrak{S}$. In a sequence of actions $e = a_1 \cdots a_n$, a send action $a_i = send(p, q, \mathbf{v})$ is *matched* by a reception $a_j = rec(p', q', \mathbf{v}')$ (denoted by $a_i \mapsto a_j$) if $i < j$, $p = p'$, $q = q'$, $\mathbf{v} = \mathbf{v}'$, and there is $\ell \geq 1$ such that $a_i$ and $a_j$ are the $\ell$th actions of $e$ with these properties respectively. A send action $a_i$ is *unmatched* if there is no matching reception in $e$. A *message exchange* of a sequence of actions $e$ is a set either of the form $v = \{a_i, a_j\}$ with $a_i \mapsto a_j$ or of the form $v = \{a_i\}$ with $a_i$ unmatched. For a message $\mathbf{v}_i$, we will note $v_i$ the corresponding message exchange. When $v$ is either an unmatched $send(p, q, \mathbf{v})$ or a pair of matched actions $\{send(p, q, \mathbf{v}), rec(p, q, \mathbf{v})\}$, we write $\mathsf{proc}_S(v)$ for $p$ and $\mathsf{proc}_R(v)$ for $q$. Note that $\mathsf{proc}_R(v)$ is defined even if $v$ is unmatched. Finally, we write $\mathsf{procs}(v)$ for $\{p\}$ in the case of an unmatched send and $\{p, q\}$ in the case of a matched send.

An execution imposes a total order on the actions. We are interested in stressing the causal dependencies between messages. We thus make use of message sequence charts (MSCs) that only impose an order between matched pairs of actions and between the actions of a same process. Informally, an MSC will be depicted with vertical timelines (one for each process) where time goes from top to bottom, that carry some events (points) representing send and receive actions of this process (see Fig. 1). An arc is drawn between two matched events. We will also draw a dashed arc to depict an unmatched send event. An MSC is, thus, a partially ordered set of events, each corresponding to a send or receive action.

**Definition 3 (MSC).** *A message sequence chart is a tuple* $(Ev, \lambda, \prec)$*, where*

- *Ev is a finite set of events,*
- $\lambda : Ev \to S \cup R$ *tags each event with an action,*
- $\prec = (\prec_{po} \cup \prec_{src})^+$ *is the transitive closure of* $\prec_{po}$ *and* $\prec_{src}$ *where:*
  - $\prec_{po}$ *is a partial order on Ev such that, for all process p,* $\prec_{po}$ *induces a total order on the set of events of process p, i.e., on* $\lambda^{-1}(S_p \cup R_p)$

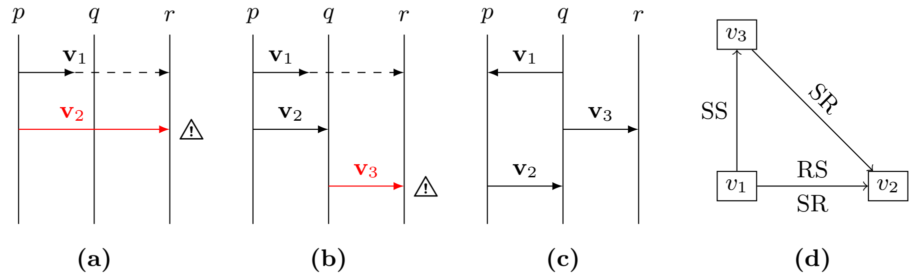

Fig. 1: (a) and (b): two MSCs that violate causal delivery. (c) and (d): an MSC and its conflict graph

- $\prec_{src}$ *is a binary relation that relates each receive event to its preceding send event :*
  - $*$ *for all events* $r \in \lambda^{-1}(R)$, *there is exactly one events* $s$ *such that* $s \prec_{src} r$
  - $*$ *for all events* $s \in \lambda^{-1}(S)$, *there is at most one event* $r$ *such that* $s \prec_{src} r$
  - $*$ *for any two events* $s, r$ *such that* $s \prec_{src} r$, *there are* $p, q, \mathbf{v}$ *such that* $\lambda(s) = send(p, q, \mathbf{v})$ *and* $\lambda(r) = rec(p, q, \mathbf{v})$.

We identify MSCs up to graph isomorphism (i.e., we view an MSC as a labeled graph). For a given *well-formed* (i.e., each reception is matched) sequence of actions $e = a_1 \dots a_n$, we let $msc(e)$ be the MSC where $Ev = [1..n]$, $\prec_{po}$ is the set of pairs of indices $(i, j)$ such that $i < j$ and $\{a_i, a_j\} \subseteq S_p \cup R_p$ for some $p \in \mathbb{P}$ (i.e., $a_i$ and $a_j$ are actions of a same process), and $\prec_{src}$ is the set of pairs of indices $(i, j)$ such that $a_i \mapsto a_j$. We say that $e = a_1 \dots a_n$ is a *linearisation* of $msc(e)$, and we write $asTr(\mathfrak{S})$ to denote $\{msc(e) \mid e \in asEx(\mathfrak{S})\}$ the set of MSCs of system $\mathfrak{S}$.

Mailbox communication imposes a number of constraints on what and when messages can be read. The precise definition is given below, we now discuss some of the possible scenarios. For instance: if two messages are sent to a same process, they will be received in the same order as they have been sent. As another example, unmatched messages also impose some constraints: if a process $p$ sends an unmatched message to $r$, it will not be able to send matched messages to $r$ afterwards (Fig. 1a); or similarly, if a process $p$ sends an unmatched message to $r$, any process $q$ that receives subsequent messages from $p$ will not be able to send matched messages to $r$ afterwards (Fig. 1b). When an MSC satisfies the constraint imposed by mailbox communication, we say that it satisfies causal delivery. Notice that, by construction, all executions satisfy causal delivery.

**Definition 4 (Causal Delivery).** *Let* $(Ev, \lambda, \prec)$ *be an MSC. We say that it satisfies causal delivery if the MSC has a linearisation* $e = a_1 \dots a_n$ *such that for any two events* $i \prec j$ *such that* $a_i = send(p, q, \mathbf{v})$ *and* $a_j = send(p', q, \mathbf{v}')$, *either* $a_j$ *is unmatched, or there are* $i', j'$ *such that* $a_i \mapsto a_{i'}$, $a_j \mapsto a_{j'}$, *and* $i' \prec j'$.

Our definition enforces the following intuitive property.

**Proposition 1.** *An MSC msc satisfies causal delivery if and only if there is a system* $\mathfrak{S}$ *and an execution* $e \in asEx(\mathfrak{S})$ *such that* $msc = msc(e)$.

We now recall from [4] the definition of *conflict graph* depicting the causal dependencies between message exchanges. Intuitively, we have a dependency whenever two messages have a process in common. For instance an $\xrightarrow{SS}$ dependency between message exchanges $v$ and $v'$ expresses the fact that $v'$ has been sent after $v$, by the same process.

**Definition 5 (Conflict Graph).** *The conflict graph* $\mathsf{CG}(e)$ *of a sequence of actions* $e = a_1 \cdots a_n$ *is the labeled graph* $(V, \{\xrightarrow{XY}\}_{X,Y\in\{R,S\}})$ *where* $V$ *is the set of message exchanges of* $e$, *and for all* $X, Y \in \{S, R\}$, *for all* $v, v' \in V$, *there is a* $XY$ *dependency edge* $v \xrightarrow{XY} v'$ *between* $v$ *and* $v'$ *if there are* $i < j$ *such that* $\{a_i\} = v \cap X$, $\{a_j\} = v' \cap Y$, *and* $\mathsf{proc}_X(v) = \mathsf{proc}_Y(v')$.

Notice that each linearisation $e$ of an MSC will have the same conflict graph. We can thus talk about an MSC and the associated conflict graph. (As an example see Figs. 1c and 1d.)

We write $v \to v'$ if $v \xrightarrow{XY} v'$ for some $X, Y \in \{R, S\}$, and $v \to^* v'$ if there is a (possibly empty) path from $v$ to $v'$.

## 3 *k*-synchronizable Systems

In this section, we define $k$-synchronizable systems. The main contribution of this part is a new characterisation of $k$-synchronizable executions that corrects the one given in [4].

In the rest of the paper, $k$ denotes a given integer $k \geq 1$. A $k$-exchange denotes a sequence of actions starting with at most $k$ sends and followed by at most $k$ receives matching some of the sends. An MSC is *k-synchronous* if there exists a linearisation that is breakable into a sequence of *k-exchanges*, such that a message sent during a $k$-exchange cannot be received during a subsequent one: either it is received during the same $k$-exchange, or it remains orphan forever.

**Definition 6 (*k*-synchronous).** *An MSC msc is k-synchronous if:*

1. *there exists a linearisation of msc* $e = e_1 \cdot e_2 \cdots e_n$ *where for all* $i \in [1..n]$, $e_i \in S^{\leq k} \cdot R^{\leq k}$,
2. *msc satisfies causal delivery,*
3. *for all* $j, j'$ *such that* $a_j \vdash\!\dashv a_{j'}$ *holds in* $e$, $a_j \vdash\!\dashv a_{j'}$ *holds in some* $e_i$.

*An execution e is k-synchronizable if* $msc(e)$ *is k-synchronous.*

We write $sTr_k(\mathfrak{S})$ to denote the set $\{msc(e) \mid e \in asEx(\mathfrak{S})$ and $msc(e)$ is $k$-synchronous$\}$.

*Example 1 (k-synchronous MSCs and k-synchronizable Executions).*

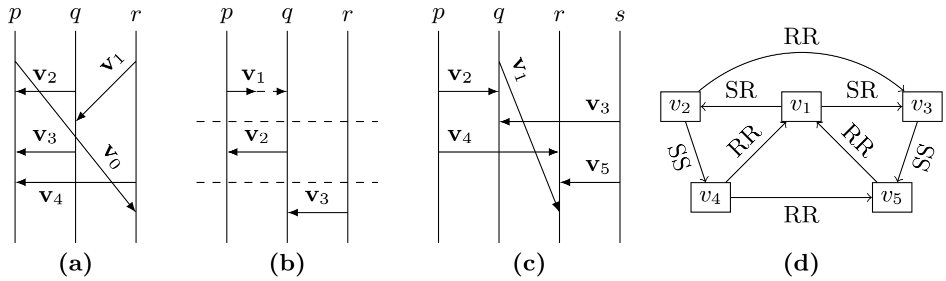

Fig. 2: (a) the MSC of Example 1.1. (b) the MSC of Example 1.2. (c) the MSC of Example 2 and (d) its conflict graph.

1. There is no $k$ such that the MSC in Fig. 2a is $k$-synchronous. All messages must be grouped in the same $k$-exchange, but it is not possible to schedule all the sends first, because the reception of $\mathbf{v}_1$ happens before the sending of $\mathbf{v}_3$. Still, this MSC satisfies causal delivery.
2. Let $e_1 = \mathit{send}(r, q, \mathbf{v}_3) \cdot \mathit{send}(q, p, \mathbf{v}_2) \cdot \mathit{send}(p, q, \mathbf{v}_1) \cdot \mathit{rec}(q, p, \mathbf{v}_2) \cdot \mathit{rec}(r, q, \mathbf{v}_3)$ be an execution. Its MSC, $\mathit{msc}(e_1)$ depicted in Fig. 2b satisfies causal delivery. Notice that $e_1$ can not be divided in 1-exchanges. However, if we consider the alternative linearisation of $\mathit{msc}(e_1)$: $e_2 = \mathit{send}(p, q, \mathbf{v}_1) \cdot \mathit{send}(q, p, \mathbf{v}_2) \cdot \mathit{rec}(q, p, \mathbf{v}_2) \cdot \mathit{send}(r, q, \mathbf{v}_3) \cdot \mathit{rec}(r, q, \mathbf{v}_3)$, we have that $e_2$ is breakable into 1-exchanges in which each matched send is in a 1-exchange with its reception. Therefore, $\mathit{msc}(e_1)$ is 1-synchronous and $e_1$ is 1-synchronizable. Remark that $e_2$ is not an execution and there exists no execution that can be divided into 1-exchanges. A $k$-synchronous MSC highlights dependencies between messages but does not impose an order for the execution.

*Comparison with [4].* In [4], the authors define set $sEx_k(\mathfrak{S})$ as the set of $k$-synchronous executions of system $\mathfrak{S}$ in the $k$-synchronous semantics. Nonetheless as remarked in Example 1.2 not all executions of a system can be divided into $k$-exchanges even if they are $k$-synchronizable. Thus, in order not to lose any executions, we have decided to reason only on MSCs (called traces in [4]).

Following standard terminology, we say that a set $U \subseteq V$ of vertices is a *strongly connected component* (SCC) of a given graph $(V, \to)$ if between any two vertices $v, v' \in U$, there exist two oriented paths $v \to^* v'$ and $v' \to^* v$. The statement below fixes some issues with Theorem 1 in [4].

**Theorem 1 (Graph Characterisation of $k$-synchronous MSCs).** *Let msc be a causal delivery MSC. msc is $k$-synchronous iff every SCC in its conflict graph is of size at most $k$ and if no RS edge occurs on any cyclic path.*

*Example 2 (A 5-synchronous MSC).* Fig. 2c depicts a 5-synchronous MSC, that is not 4-synchronous. Indeed, its conflict graph (Fig. 2d) contains a SCC of size 5 (all vertices are on the same SCC).

*Comparison with [4].* Bouajjani *et al.* give a characterisation of $k$-synchronous executions similar to ours, but they use the word *cycle* instead of SCC, and the subsequent developments of the paper suggest that they intended to say *Hamiltonian cycle* (i.e., a cyclic path that does not go twice through the same vertex). It is not the case that a MSC is $k$-synchronous if and only if every Hamiltonian cycle in its conflict graph is of size at most $k$ and if no RS edge occurs on any cyclic path. Indeed, consider again Example 2. This graph is not Hamiltonian, and the largest Hamiltonian cycle indeed is of size 4 only. But as we already discussed in Example 2, the corresponding MSC is not 4-synchronous.

As a consequence, the algorithm that is presented in [4] for deciding whether a system is $k$-synchronizable is not correct as well: the MSC of Fig. 2c would be considered 4-synchronous according to this algorithm, but it is not.

## 4 Decidability of Reachability for *k*-synchronizable Systems

We show that the reachability problem is decidable for $k$-synchronizable systems. While proving this result, we have to face several non-trivial aspects of causal delivery that were missed in [4] and that require a completely new approach.

**Definition 7 ($k$-synchronizable System).** *A system $\mathfrak{S}$ is $k$-*synchronizable *if all its executions are $k$-synchronizable, i.e., $sTr_k(\mathfrak{S}) = asTr(\mathfrak{S})$.*

In other words, a system $\mathfrak{S}$ is $k$-synchronizable if for every execution $e$ of $\mathfrak{S}$, $msc(e)$ may be divided into $k$-exchanges.

*Remark 1.* In particular, a system may be $k$-synchronizable even if some of its executions fill the buffers with more than $k$ messages. For instance, the only linearisation of the 1-synchronous MSC Fig. 2b that is an execution of the system needs buffers of size 2.

For a $k$-synchronizable system, the reachability problem reduces to the reachability through a $k$-synchronizable execution. To show that $k$-synchronous reachability is decidable, we establish that the set of $k$-synchronous MSCs is regular. More precisely, we want to define a finite state automaton that accepts a sequence $e_1 \cdot e_2 \cdots e_n$ of $k$-exchanges if and only if they satisfy causal delivery.

We start by giving a graph-theoretic characterisation of causal delivery. For this, we define the *extended edges* $v \overset{XY}{\dashrightarrow} v'$ of a given conflict graph. The relation $\overset{XY}{\dashrightarrow}$ is defined in Fig. 3 with $X, Y \in \{S, R\}$. Intuitively, $v \overset{XY}{\dashrightarrow} v'$ expresses that event $X$ of $v$ must happen before event $Y$ of $v'$ due to either their order on the same machine (Rule 1), or the fact that a send happens before its matching receive (Rule 2), or due to the mailbox semantics (Rules 3 and 4), or because of a chain of such dependencies (Rule 5). We observe that in the extended *conflict graph*, obtained applying such rules, a cyclic dependency appears whenever causal delivery is not satisfied.

$$\text{(Rule 1)}\ \frac{v_1 \xrightarrow{XY} v_2}{v_1 \overset{XY}{\dashrightarrow} v_2} \qquad \text{(Rule 2)}\ \frac{v \cap R \neq \emptyset}{v \overset{SR}{\dashrightarrow} v} \qquad \text{(Rule 3)}\ \frac{v_1 \xrightarrow{RR} v_2}{v_1 \overset{SS}{\dashrightarrow} v_2}$$

$$\text{(Rule 4)}\ \frac{v_1 \cap R \neq \emptyset \qquad v_2 \cap R = \emptyset \qquad \mathsf{proc}_R(v_1) = \mathsf{proc}_R(v_2)}{v_1 \overset{SS}{\dashrightarrow} v_2} \qquad \text{(Rule 5)}\ \frac{v_1 \overset{XY}{\dashrightarrow}\overset{YZ}{\dashrightarrow} v_2}{v_1 \overset{XZ}{\dashrightarrow} v_2}$$

Fig. 3: Deduction rules for extended dependency edges of the conflict graph

*Example 3.* Fig. 5a and 5b depict an MSC and its associated conflict graph with some extended edges. This MSC violates causal delivery and there is a cyclic dependency $v_1 \overset{SS}{\dashrightarrow} v_1$.

**Theorem 2 (Graph-theoretic Characterisation of Causal Delivery).** *An MSC satisfies causal delivery iff there is no cyclic causal dependency of the form* $v \overset{SS}{\dashrightarrow} v$ *for some vertex* $v$ *of its extended conflict graph.*

Let us now come back to our initial problem: we want to recognise with finite memory the sequences $e_1, e_2 \ldots e_n$ of $k$-exchanges that composed give an MSC that satisfies causal delivery. We proceed by reading each $k$-exchange one by one in sequence. This entails that, at each step, we have only a partial view of the global conflict graph. Still, we want to determine whether the acyclicity condition of Theorem 2 is satisfied in the global conflict graph. The crucial observation is that only the edges generated by Rule 4 may "go back in time". This means that we have to remember enough information from the previously examined $k$-exchanges to determine whether the current $k$-exchange contains a vertex $v$ that shares an edge with some unmatched vertex $v'$ seen in a previous $k$-exchange and whether this could participate in a cycle. This is achieved by computing two sets of processes $C_{S,p}$ and $C_{R,p}$ that collect the following information: a process $q$ is in $C_{S,p}$ if it performs a send action causally after an unmatched send to $p$, or it is the sender of the unmatched send; a process $q$ belongs to $C_{R,p}$ if it receives a message that was sent after some unmatched message directed to $p$. More precisely, we have:

$$C_{S,p} = \{\mathsf{proc}_S(v) \mid v' \overset{SS}{\dashrightarrow} v \ \&\ v' \text{ is unmatched } \&\ \mathsf{proc}_R(v') = p\}$$

$$C_{R,p} = \{\mathsf{proc}_R(v) \mid v' \overset{SS}{\dashrightarrow} v \ \&\ v' \text{ is unmatched } \&\ \mathsf{proc}_R(v') = p \ \&\ v \cap R \neq \emptyset\}$$

These sets abstract and carry from one $k$-exchange to another the necessary information to detect violations of causal delivery. We compute them in any local conflict graph of a $k$-exchange incrementally, i.e., knowing what they were at the end of the previous $k$-exchange, we compute them at the end of the current one. More precisely, let $e = s_1 \cdots s_m \cdot r_1 \cdots r_{m'}$ be a $k$-exchange, $\mathsf{CG}(e) = (V, E)$ its conflict graph and $B : \mathbb{P} \to (2^{\mathbb{P}} \times 2^{\mathbb{P}})$ the function that associates to each $p \in \mathbb{P}$ the two sets $B(p) = (C_{S,p}, C_{R,p})$. Then, the conflict graph $\mathsf{CG}(e, B)$ is the graph $(V', E')$ with $V' = V \cup \{\psi_p \mid p \in \mathbb{P}\}$ and $E' \supseteq E$ as defined below. For each process $p \in \mathbb{P}$, the "summary node" $\psi_p$ shall account for all past unmatched

$$\frac{\begin{array}{c} e = s_1 \cdots s_m \cdot r_1 \cdots r_{m'} \quad s_1 \cdots s_m \in S^* \quad r_1 \cdots r_{m'} \in R^* \quad 0 \leq m' \leq m \leq k \\ (\vec{l}, \mathtt{Buf}_0) \overset{e}{\Rightarrow} (\vec{l'}, \mathtt{Buf}) \text{ for some } \mathtt{Buf} \\ \text{for all } p \in \mathbb{P} \quad B(p) = (C_{S,p}, C_{R,p}) \text{ and } B'(p) = (C'_{S,p}, C'_{R,p}), \\ \mathsf{Unm}_p = \{\psi_p\} \cup \{v \mid v \text{ is unmatched, } \mathsf{proc}_R(v) = p\} \\ C'_{X,p} = C_{X,p} \cup \{p \mid p \in C_{X,q}, v \overset{SS}{\dashrightarrow} \psi_q, (\mathsf{proc}_R(v) = p \text{ or } v = \psi_p)\} \cup \\ \{\mathsf{proc}_X(v) \mid v \in \mathsf{Unm}_p \cap V, X = S\} \cup \{\mathsf{proc}_X(v') \mid v \overset{SS}{\dashrightarrow} v', v \in \mathsf{Unm}_p, v \cap X \neq \emptyset\} \\ \text{for all } p \in \mathbb{P}, p \notin C'_{R,p} \end{array}}{(\vec{l}, B) \underset{\mathsf{cd}}{\overset{e,k}{\Longrightarrow}} (\vec{l'}, B')}$$

Fig. 4: Definition of the relation $\underset{\mathsf{cd}}{\overset{e,k}{\Longrightarrow}}$

messages sent to $p$ that occurred in some $k$-exchange before $e$. $E'$ is the set $E$ of edges $\overset{XY}{\longrightarrow}$ among message exchanges of $e$, as in Definition 5, augmented with the following set of extra edges that takes into account summary nodes.

$$\{\psi_p \overset{SX}{\longrightarrow} v \mid \mathsf{proc}_X(v) \in C_{S,p} \ \&\ v \cap X \neq \emptyset \text{ for some } X \in \{S, R\}\} \tag{1}$$
$$\cup\ \{\psi_p \overset{SS}{\longrightarrow} v \mid \mathsf{proc}_X(v) \in C_{R,p} \ \&\ v \cap R \neq \emptyset \text{ for some } X \in \{S, R\}\} \tag{2}$$
$$\cup\ \{\psi_p \overset{SS}{\longrightarrow} v \mid \mathsf{proc}_R(v) \in C_{R,p} \ \&\ v \text{ is unmatched}\} \tag{3}$$
$$\cup\ \{v \overset{SS}{\longrightarrow} \psi_p \mid \mathsf{proc}_R(v) = p \ \&\ v \cap R \neq \emptyset\} \ \cup\ \{\psi_q \overset{SS}{\longrightarrow} \psi_p \mid p \in C_{R,q}\} \tag{4}$$

These extra edges summarise/abstract the connections to and from previous $k$-exchanges. Equation (1) considers connections $\overset{SS}{\longrightarrow}$ and $\overset{SR}{\longrightarrow}$ that are due to two sends messages or, respectively, a send and a receive on the same process. Equations (2) and (3) considers connections $\overset{RR}{\longrightarrow}$ and $\overset{RS}{\longrightarrow}$ that are due to two received messages or, respectively, a receive and a subsequent send on the same process. Notice how the rules in Fig. 3 would then imply the existence of a connection $\overset{SS}{\dashrightarrow}$, in particular Equation (3) abstract the existence of an edge $\overset{SS}{\dashrightarrow}$ built because of Rule 4. Equations in (4) abstract edges that would connect the current $k$-exchange to previous ones. As before those edges in the global conflict graph would correspond to extended edges added because of Rule 4 in Fig. 3. Once we have this enriched local view of the conflict graph, we take its extended version. Let $\overset{XY}{\dashrightarrow}$ denote the edges of the extended conflict graph as defined from rules in Fig. 3 taking into account the new vertices $\psi_p$ and their edges.

Finally, let $\mathfrak{S}$ be a system and $\underset{\mathsf{cd}}{\overset{e,k}{\Longrightarrow}}$ be the transition relation given in Fig. 4 among abstract configurations of the form $(\vec{l}, B)$. $\vec{l}$ is a global control state of $\mathfrak{S}$ and $B : \mathbb{P} \to \left(2^{\mathbb{P}} \times 2^{\mathbb{P}}\right)$ is the function defined above that associates to each process $p$ a pair of sets of processes $B(p) = (C_{S,p}, C_{R,p})$. Transition $\underset{\mathsf{cd}}{\overset{e,k}{\Longrightarrow}}$ updates these sets with respect to the current $k$-exchange $e$. Causal delivery is verified by checking that for all $p \in \mathbb{P}, p \notin C'_{R,p}$ meaning that there is no cyclic dependency

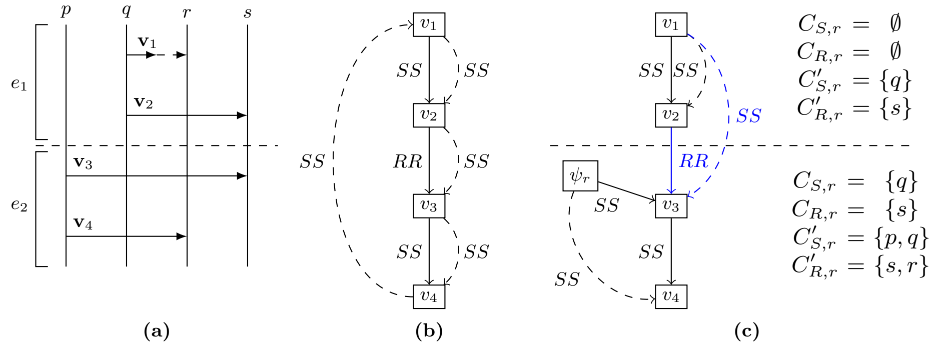

Fig. 5: (a) an MSC (b) its associated global conflict graph, (c) the conflict graphs of its $k$-exchanges as stated in Theorem 2. The initial state is $(\vec{l_0}, B_0)$, where $B_0 : \mathbb{P} \to (2^{\mathbb{P}} \times 2^{\mathbb{P}})$ denotes the function such that $B_0(p) = (\emptyset, \emptyset)$ for all $p \in \mathbb{P}$.

*Example 4 (An Invalid Execution).* Let $e = e_1 \cdot e_2$ with $e_1$ and $e_2$ the two 2-exchanges of this execution. such that $e_1 = send(q, r, \mathbf{v}_1) \cdot send(q, s, \mathbf{v}_2) \cdot rec(q, s, \mathbf{v}_2)$ and $e_2 = send(p, s, \mathbf{v}_3) \cdot rec(p, s, \mathbf{v}_3) \cdot send(p, r, \mathbf{v}_4) \cdot rec(p, r, \mathbf{v}_4)$. Fig. 5a and 5c show the MSC and corresponding conflict graph of each of the 2-exchanges. Note that two edges of the global graph (in blue) "go across" $k$-exchanges. These edges do not belong to the local conflict graphs and are mimicked by the incoming and outgoing edges of summary nodes. The values of sets $C_{S,r}$ and $C_{R,r}$ at the beginning and at the end of the $k$-exchange are given on the right. All other sets $C_{S,p}$ and $C_{R,p}$ for $p \neq r$ are empty, since there is only one unmatched message to process $r$. Notice how at the end of the second $k$-exchange, $r \in C'_{R,r}$ signalling that message $v_4$ violates causal delivery.

*Comparison with [4].* In [4] the authors define $\xRightarrow[\mathsf{cd}]{e,k}$ in a rather different way: they do not explicitly give a graph-theoretic characterisation of causal delivery; instead they compute, for every process $p$, the set $B(p)$ of processes that either sent an unmatched message to $p$ or received a message from a process in $B(p)$. They then make sure that any message sent to $p$ by a process $q \in B(p)$ is unmatched. According to that definition, the MSC of Fig. 5b would satisfy causal delivery and would be 1-synchronous. However, this is not the case (this MSC does not satisfy causal delivery) as we have shown in Example 3. Due to to the above errors, we had to propose a considerably different approach. The extended edges of the conflict graph, and the graph-theoretic characterisation of causal delivery as well as summary nodes, have no equivalent in [4].

Next lemma proves that Fig. 4 properly characterises causal delivery.

**Lemma 1.** *An MSC msc is $k$-synchronous iff there is $e = e_1 \cdots e_n$ a linearisation such that $(\vec{l_0}, B_0) \xRightarrow[\mathsf{cd}]{e_1,k} \cdots \xRightarrow[\mathsf{cd}]{e_n,k} (\vec{l'}, B')$ for some global state $\vec{l'}$ and some $B' : \mathbb{P} \to (2^{\mathbb{P}} \times 2^{\mathbb{P}})$.*

Note that there are only finitely many abstract configurations of the form $(\vec{l}, B)$ with $\vec{l}$ a tuple of control states and $B : \mathbb{P} \to (2^{\mathbb{P}} \times 2^{\mathbb{P}})$. Moreover, since $\mathbb{V}$ is finite, the alphabet over the possible $k$-exchange for a given $k$ is also finite. Therefore $\xRightarrow[\mathsf{cd}]{e,k}$ is a relation on a finite set, and the set $sTr_k(\mathfrak{S})$ of $k$-synchronous MSCs of a system $\mathfrak{S}$ forms a regular language. It follows that it is decidable whether a given abstract configuration of the form $(\vec{l}, B)$ is reachable from the initial configuration following a $k$-synchronizable execution.

**Theorem 3.** *Let $\mathfrak{S}$ be a $k$-synchronizable system and $\vec{l}$ a global control state of $\mathfrak{S}$. The problem whether there exists $e \in asEx(\mathfrak{S})$ and $\mathtt{Buf}$ such that $(\vec{l_0}, \mathtt{Buf}_0) \xRightarrow{e} (\vec{l}, \mathtt{Buf})$ is decidable.*

*Remark 2.* Deadlock-freedom, unspecified receptions, and absence of orphan messages are other properties that become decidable for a $k$-synchronizable system because of the regularity of the set of $k$-synchronous MSCs.

## 5 Decidability of $k$-synchronizability for Mailbox Systems

We establish the decidability of $k$-synchronizability; our approach is similar to the one of [4] based on the notion of borderline violation, but we adjust it to adapt to the new characterisation of $k$-synchronizable executions (Theorem 1).

**Definition 8 (Borderline Violation).** *A non $k$-synchronizable execution $e$ is a borderline violation if $e = e' \cdot r$, $r$ is a reception and $e'$ is $k$-synchronizable.*

Note that a system $\mathfrak{S}$ that is not $k$-synchronizable always admits at least one borderline violation $e' \cdot r \in asEx(\mathfrak{S})$ with $r \in R$: indeed, there is at least one execution $e \in asEx(\mathfrak{S})$ which contains a unique minimal prefix of the form $e' \cdot r$ that is not $k$-synchronizable; moreover since $e'$ is $k$-synchronizable, $r$ cannot be a $k$-exchange of just one send action, therefore it must be a receive action. In order to find such a borderline violation, Bouajjani *et al.* introduced an instrumented system $\mathfrak{S}'$ that behaves like $\mathfrak{S}$, except that it contains an extra process $\pi$, and such that a non-deterministically chosen message that should have been sent from a process $p$ to a process $q$ may now be sent from $p$ to $\pi$, and later forwarded by $\pi$ to $q$. In $\mathfrak{S}'$, each process $p$ has the possibility, instead of sending a message $\mathbf{v}$ to $q$, to deviate this message to $\pi$; if it does so, $p$ continues its execution as if it really had sent it to $q$. Note also that the message sent to $\pi$ get tagged with the original destination process $q$. Similarly, for each possible reception, a process has the possibility to receive a given message not from the initial sender but from $\pi$. The process $\pi$ has an initial state from which it can receive any messages from the system. Each reception makes it go into a different state. From this state, it is able to send the message back to the original recipient. Once a message is forwarded, $\pi$ reaches its final state and remains idle. The following example illustrates how the instrumented system works.

*Example 5 (A Deviated Message).*
Let $e_1$, $e_2$ be two executions of a system $\mathfrak{S}$ with MSCs respectively $msc(e_1)$ and $msc(e_2)$. $e_1$ is not 1-synchronizable. It is borderline in $\mathfrak{S}$. If we delete the last reception, it becomes indeed 1-synchronizable. $msc(e_2)$ is the MSC obtained from the instrumented system $\mathfrak{S}'$ where the message $\mathbf{v}_1$ is first deviated to $\pi$ and then sent back to $q$ from $\pi$.
Note that $msc(e_2)$ is 1-synchronous. In this case, the instrumented system $\mathfrak{S}'$ in the 1-synchronous semantics "reveals" the existence of a borderline violation of $\mathfrak{S}$.

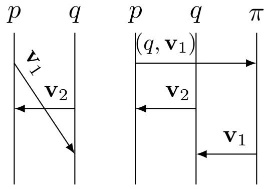

$msc(e_1)$ $msc(e_2)$

For each execution $e \cdot r \in asEx(\mathfrak{S})$ that ends with a reception, there exists an execution $\mathsf{deviate}(e \cdot r) \in asEx(\mathfrak{S}')$ where the message exchange associated with the reception $r$ has been deviated to $\pi$; formally, if $e \cdot r = e_1 \cdot s \cdot e_2 \cdot r$ with $r = rec(p, q, \mathbf{v})$ and $s \vdash\!\dashv r$, then

$$\mathsf{deviate}(e{\cdot}r) = e_1{\cdot}send(p, \pi, (q, \mathbf{v})){\cdot}rec(p, \pi, (q, \mathbf{v})){\cdot}e_2{\cdot}send(\pi, q, (\mathbf{v})){\cdot}rec(\pi, q, \mathbf{v}).$$

**Definition 9 (Feasible Execution, Bad Execution).** *A $k$-synchronizable execution $e'$ of $\mathfrak{S}'$ is* feasible *if there is an execution $e \cdot r \in asEx(\mathfrak{S})$ such that* $\mathsf{deviate}(e{\cdot}r) = e'$. *A feasible execution $e' = \mathsf{deviate}(e{\cdot}r)$ of $\mathfrak{S}'$ is* bad *if execution $e \cdot r$ is not $k$-synchronizable in $\mathfrak{S}$.*

*Example 6 (A Non-feasible Execution).*
Let $e'$ be an execution such that $msc(e')$ is as depicted on the right. Clearly, this MSC satisfies causal delivery and could be the execution of some instrumented system $\mathfrak{S}'$. However, the sequence $e{\cdot}r$ such that $\mathsf{deviate}(e{\cdot}r) = e'$ does not satisfy causal delivery, therefore it cannot be an execution of the original system $\mathfrak{S}$. In other words, the execution $e'$ is not feasible.

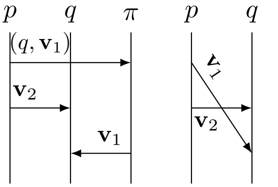

$msc(e')$ $msc(e \cdot r)$

**Lemma 2.** *A system $\mathfrak{S}$ is not $k$-synchronizable iff there is a $k$-synchronizable execution $e'$ of $\mathfrak{S}'$ that is feasible and bad.*

As we have already noted, the set of $k$-synchronous MSCs of $\mathfrak{S}'$ is regular. The decision procedure for $k$-synchronizability follows from the fact that the set of MSCs that have as linearisation a feasible bad execution as we will see, is regular as well, and that it can be recognised by an (effectively computable) non-deterministic finite state automaton. The decidability of $k$-synchronizability follows then from Lemma 2 and the decidability of the emptiness problem for non-deterministic finite state automata.

**Recognition of Feasible Executions.** We start with the automaton that recognises feasible executions; for this, we revisit the construction we just used for recognising sequences of $k$-exchanges that satisfy causal delivery.

In the remainder, we assume an execution $e' \in asEx(\mathfrak{S}')$ that contains exactly one send of the form $send(p, \pi, (q, \mathbf{v}))$ and one reception of the form $rec(\pi, q, \mathbf{v})$, this reception being the last action of $e'$. Let $(V, \{\xrightarrow{XY}\}_{X,Y \in \{R,S\}})$ be the conflict graph of $e'$. There are two uniquely determined vertices $\upsilon_{\mathsf{start}}, \upsilon_{\mathsf{stop}} \in V$ such that $\mathsf{proc}_R(\upsilon_{\mathsf{start}}) = \pi$ and $\mathsf{proc}_S(\upsilon_{\mathsf{stop}}) = \pi$ that correspond, respectively, to the first and last message exchanges of the deviation. The conflict graph of $e \cdot r$ is then obtained by merging these two nodes.

**Lemma 3.** *The execution $e'$ is not feasible iff there is a vertex $v$ in the conflict graph of $e'$ such that $\upsilon_{\mathsf{start}} \overset{SS}{\dashrightarrow} v \xrightarrow{RR} \upsilon_{\mathsf{stop}}$.*

In order to decide whether an execution $e'$ is feasible, we want to forbid that a send action $send(p', q, \mathbf{v}')$ that happens causally after $\upsilon_{\mathsf{start}}$ is matched by a receive $rec(p', q, \mathbf{v}')$ that happens causally before the reception $\upsilon_{\mathsf{stop}}$. As a matter of fact, this boils down to deal with the deviated send action as an unmatched send. So we will consider sets of processes $C_S^\pi$ and $C_R^\pi$ similar to the ones used for $\overset{e,k}{\underset{\mathsf{cd}}{\Longrightarrow}}$, but with the goal of computing which actions happen causally after the send to $\pi$. We also introduce a summary node $\psi_{\mathsf{start}}$ and the extra edges following the same principles as in the previous section. Formally, let $B : \mathbb{P} \to (2^{\mathbb{P}} \times 2^{\mathbb{P}})$, $C_S^\pi, C_R^\pi \subseteq \mathbb{P}$ and $e \in S^{\leq k}R^{\leq k}$ be fixed, and let $\mathsf{CG}(e, B) = (V', E')$ be the constraint graph with summary nodes for unmatched sent messages as defined in the previous section. The local constraint graph $\mathsf{CG}(e, B, C_S^\pi, C_R^\pi)$ is defined as the graph $(V'', E'')$ where $V'' = V' \cup \{\psi_{\mathsf{start}}\}$ and $E''$ is $E'$ augmented with

$$\{\psi_{\mathsf{start}} \xrightarrow{SX} v \mid \mathsf{proc}_X(v) \in C_S^\pi \ \&\ v \cap X \neq \emptyset \text{ for some } X \in \{S, R\}\}$$
$$\cup\ \{\psi_{\mathsf{start}} \xrightarrow{SS} v \mid \mathsf{proc}_X(v) \in C_R^\pi \ \&\ v \cap R \neq \emptyset \text{ for some } X \in \{S, R\}\}$$
$$\cup\ \{\psi_{\mathsf{start}} \xrightarrow{SS} v \mid \mathsf{proc}_R(v) \in C_R^\pi \ \&\ v \text{ is unmatched}\} \ \cup\ \{\psi_{\mathsf{start}} \xrightarrow{SS} \psi_p \mid p \in C_R^\pi\}$$

As before, we consider the "closure" $\overset{XY}{\dashrightarrow}$ of these edges by the rules of Fig. 3. The transition relation $\overset{e,k}{\underset{\mathsf{feas}}{\Longrightarrow}}$ is defined in Fig. 6. It relates abstract configurations of the form $(\vec{l}, B, \vec{C}, \mathtt{dest}_\pi)$ with $\vec{C} = (C_{S,\pi}, C_{R,\pi})$ and $\mathtt{dest}_\pi \in \mathbb{P} \cup \{\bot\}$ storing to whom the message deviated to $\pi$ was supposed to be delivered. Thus, the initial abstract configuration is $(l_0, B_0, (\emptyset, \emptyset), \bot)$, where $\bot$ means that the processus $\mathtt{dest}_\pi$ has not been determined yet. It will be set as soon as the send to process $\pi$ is encountered.

**Lemma 4.** *Let $e'$ be an execution of $\mathfrak{S}'$. Then $e'$ is a $k$-synchronizable feasible execution iff there are $e'' = e_1 \cdots e_n \cdot send(\pi, q, \mathbf{v}) \cdot rec(\pi, q, \mathbf{v})$ with $e_1, \ldots, e_n \in S^{\leq k}R^{\leq k}$, $B' : \mathbb{P} \to 2^{\mathbb{P}}$, $\vec{C'} \in (2^{\mathbb{P}})^2$, and a tuple of control states $\vec{l'}$ such that $msc(e') = msc(e'')$, $\pi \notin C_{R,q}$ (with $B'(q) = (C_{S,q}, C_{R,q})$), and*

$$(\vec{l_0}, B_0, (\emptyset, \emptyset), \bot) \overset{e_1,k}{\underset{\mathsf{feas}}{\Longrightarrow}} \ldots \overset{e_n,k}{\underset{\mathsf{feas}}{\Longrightarrow}} (\vec{l'}, B', \vec{C'}, q).$$

$$\frac{\begin{array}{c}(\vec{l}, B) \xRightarrow[\mathsf{cd}]{e,k} (\vec{l'}, B') \qquad e = a_1 \cdots a_n \qquad (\forall v)\ \mathsf{proc}_S(v) \neq \pi \\ (\forall v, v')\ \mathsf{proc}_R(v) = \mathsf{proc}_R(v') = \pi \implies v = v' \wedge \mathtt{dest}_\pi = \bot \\ (\forall v)\ v \ni \mathit{send}(p, \pi, (q, \mathbf{v})) \implies \mathtt{dest}'_\pi = q \quad \mathtt{dest}_\pi \neq \bot \implies \mathtt{dest}'_\pi = \mathtt{dest}_\pi \\ C^\pi_X{}' = C^\pi_X \cup \{\mathsf{proc}_X(v') \mid v \overset{SS}{\dashrightarrow} v' \ \&\ v' \cap X \neq \emptyset \ \&\ (\mathsf{proc}_R(v) = \pi \text{ or } v = \psi_{\mathsf{start}})\} \\ \cup \{\mathsf{proc}_S(v) \mid \mathsf{proc}_R(v) = \pi \ \&\ X = S\} \\ \cup \{p \mid p \in C_{X,q} \ \&\ v \overset{SS}{\dashrightarrow} \psi_q \ \&\ (\mathsf{proc}_R(v) = \pi \text{ or } v = \psi_{\mathsf{start}})\} \\ \mathtt{dest}'_\pi \notin C^\pi_R{}'\end{array}}{(\vec{l}, B, C^\pi_S, C^\pi_R, \mathtt{dest}_\pi) \xRightarrow[\mathsf{feas}]{e,k} (\vec{l'}, B', C^\pi_S{}', C^\pi_R{}', \mathtt{dest}'_\pi)}$$

Fig. 6: Definition of the relation $\xRightarrow[\mathsf{feas}]{e,k}$

*Comparison with [4].* In [4] the authors verify that an execution is feasible with a *monitor* which reviews the actions of the execution and adds processes that no longer are allowed to send a message to the receiver of $\pi$. Unfortunately, we have here a similar problem that the one mentioned in the previous comparison paragraph. According to their monitor, the following execution $e' = \mathsf{deviate}(e \cdot r)$ is feasible, i.e., is runnable in $\mathfrak{S}'$ and $e \cdot r$ is runnable in $\mathfrak{S}$.

$$\begin{aligned} e' = \ & \mathit{send}(q, \pi, (r, \mathbf{v}_1)) \cdot \mathit{rec}(q, \pi, (r, \mathbf{v}_1)) \cdot \mathit{send}(q, s, \mathbf{v}_2) \cdot \mathit{rec}(q, s, \mathbf{v}_2) \cdot \\ & \mathit{send}(p, s, \mathbf{v}_3) \cdot \mathit{rec}(p, s, \mathbf{v}_3) \cdot \mathit{send}(p, r, \mathbf{v}_4) \cdot \mathit{rec}(p, r, \mathbf{v}_4) \cdot \\ & \mathit{send}(\pi, r, \mathbf{v}_1) \cdot \mathit{rec}(\pi, r, \mathbf{v}_4) \end{aligned}$$

However, this execution is not feasible because there is a causal dependency between $\mathbf{v}_1$ and $\mathbf{v}_3$. In [4] this execution would then be considered as feasible and therefore would belong to set $sTr_k(\mathfrak{S}')$. Yet there is no corresponding execution in $asTr(\mathfrak{S})$, the comparison and therefore the $k$-synchronizability, could be distorted and appear as a false negative.

**Recognition of Bad Executions.** Finally, we define a non-deterministic finite state automaton that recognizes MSCs of bad executions, i.e., feasible executions $e' = \mathsf{deviate}(e \cdot r)$ such that $e \cdot r$ is not $k$-synchronizable. We come back to the "non-extended" conflict graph, without edges of the form $\overset{XY}{\dashrightarrow}$. Let $\mathsf{Post}^*(v) = \{v' \in V \mid v \to^* v'\}$ be the set of vertices reachable from $v$, and let $\mathsf{Pre}^*(v) = \{v' \in V \mid v' \to^* v\}$ be the set of vertices co-reachable from $v$. For a set of vertices $U \subseteq V$, let $\mathsf{Post}^*(U) = \bigcup\{\mathsf{Post}^*(v) \mid v \in U\}$, and $\mathsf{Pre}^*(U) = \bigcup\{\mathsf{Pre}^*(v) \mid v \in U\}$.

**Lemma 5.** *The feasible execution $e'$ is bad iff one of the two holds*

1. $v_{\mathsf{start}} \longrightarrow^* \xrightarrow{RS} \longrightarrow^* v_{\mathsf{stop}}$, *or*
2. *the size of the set* $\mathsf{Post}^*(v_{\mathsf{start}}) \cap \mathsf{Pre}^*(v_{\mathsf{stop}})$ *is greater or equal to* $k + 2$.

In order to determine whether a given message exchange $v$ of $\mathsf{CG}(e')$ should be counted as reachable (resp. co-reachable), we will compute at the entry and exit of every $k$-exchange of $e'$ which processes are "reachable" or "co-reachable".

*Example 7.* (Reachable and Co-reachable Processes)

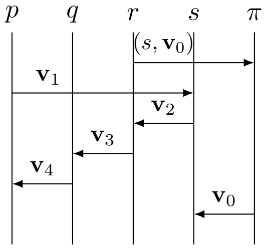

$msc(e)$

Consider the MSC on the right made of five 1-exchanges. While sending message $(s, \mathbf{v}_0)$ that corresponds to $v_{\mathsf{start}}$, process $r$ becomes "reachable": any subsequent message exchange that involves $r$ corresponds to a vertex of the conflict graph that is reachable from $v_{\mathsf{start}}$. While sending $\mathbf{v}_2$, process $s$ becomes "reachable", because process $r$ will be reachable when it will receive message $\mathbf{v}_2$. Similary, $q$ becomes reachable after receiving $\mathbf{v}_3$ because $r$ was reachable when it sent $\mathbf{v}_3$, and $p$ becomes reachable after receiving $\mathbf{v}_4$ because $q$ was reachable when it sent $\mathbf{v}_4$. Co-reachability works similarly, but reasoning backwards on the timelines. For instance, process $s$ stops being "co-reachable" while it receives $\mathbf{v}_0$, process $r$ stops being co-reachable after it receives $\mathbf{v}_2$, and process $p$ stops being co-reachable by sending $\mathbf{v}_1$. The only message that is sent by a process being both reachable and co-reachable at the instant of the sending is $\mathbf{v}_2$, therefore it is the only message that will be counted as contributing to the SCC.

More formally, let $e$ be sequence of actions, $\mathsf{CG}(e)$ its conflict graph and $P, Q$ two sets of processes, $\mathsf{Post}_e(P) = \mathsf{Post}^*\Big(\{v \mid \mathsf{procs}(v) \cap P \neq \emptyset\}\Big)$ and $\mathsf{Pre}_e(Q) = \mathsf{Pre}^*\Big(\{v \mid \mathsf{procs}(v) \cap Q \neq \emptyset\}\Big)$ are introduced to represent the local view through $k$-exchanges of $\mathsf{Post}^*(v_{\mathsf{start}})$ and $\mathsf{Pre}^*(v_{\mathsf{stop}})$. For instance, for $e$ as in Example 7, we get $\mathsf{Post}_e(\{\pi\}) = \{(s, \mathbf{v}_0), \mathbf{v}_2, \mathbf{v}_3, \mathbf{v}_4, \mathbf{v}_0\}$ and $\mathsf{Pre}_e(\{\pi\}) = \{\mathbf{v}_0, \mathbf{v}_2, \mathbf{v}_1, (s, \mathbf{v}_0)\}$. In each $k$-exchange $e_i$ the size of the intersection between $\mathsf{Post}_{e_i}(P)$ and $\mathsf{Pre}_{e_i}(Q)$ will give the local contribution of the current $k$-exchange to the calculation of the size of the global SCC. In the transition relation $\xrightarrow[\mathsf{bad}]{e,k}$ this value is stored in variable $\mathtt{cnt}$. The last ingredient to consider is to recognise if an edge RS belongs to the SCC. To this aim, we use a function $\mathtt{lastisRec} : \mathbb{P} \to \{\mathsf{True}, \mathsf{False}\}$ that for each process stores the information whether the last action in the previous $k$-exchange was a reception or not. Then depending on the value of this variable and if a node is in the current SCC or not the value of $\mathtt{sawRS}$ is set accordingly.

The transition relation $\xrightarrow[\mathsf{bad}]{e,k}$ defined in Fig. 7 deals with abstract configurations of the form $(P, Q, \mathtt{cnt}, \mathtt{sawRS}, \mathtt{lastisRec}')$ where $P, Q \subseteq \mathbb{P}$, $\mathtt{sawRS}$ is a boolean value, and $\mathtt{cnt}$ is a counter bounded by $k+2$. We denote by $\mathtt{lastisRec}_0$ the function where all $\mathtt{lastisRec}(p) = \mathsf{False}$ for all $p \in \mathbb{P}$.

**Lemma 6.** *Let $e'$ be a feasible $k$-synchronizable execution of $\mathfrak{S}'$. Then $e'$ is a bad execution iff there are $e'' = e_1 \cdots e_n \cdot send(\pi, q, \mathbf{v}) \cdot rec(\pi, q, \mathbf{v})$ with $e_1, \ldots, e_n \in S^{\leq k} R^{\leq k}$ and $msc(e') = msc(e'')$, $P', Q \subseteq \mathbb{P}$, $\mathtt{sawRS} \in \{\mathsf{True}, \mathsf{False}\}$, $\mathtt{cnt} \in \{0, \ldots, k+2\}$, such that*

$$(\{\pi\}, Q, 0, \mathsf{False}, \mathtt{lastisRec}_0) \xrightarrow[\mathsf{bad}]{e_1,k} \ldots \xrightarrow[\mathsf{bad}]{e_n,k} (P', \{\pi\}, \mathtt{cnt}, \mathtt{sawRS}, \mathtt{lastisRec})$$

$$\frac{\begin{array}{c} P' = \mathsf{procs}(\mathsf{Post}_e(P)) \qquad Q = \mathsf{procs}(\mathsf{Pre}_e(Q')) \\ SCC_e = \mathsf{Post}_e(P) \cap \mathsf{Pre}_e(Q') \\ \mathtt{cnt}' = \mathsf{min}(k+2, \mathtt{cnt}+n) \quad \text{where } n = |SCC_e| \\ \mathtt{lastisRec}'(q) \Leftrightarrow (\exists v \in SCC_e.\mathsf{proc}_R(v) = q \wedge v \cap R \neq \emptyset) \vee \\ (\mathtt{lastisRec}(q) \wedge \nexists v \in V.\mathsf{proc}_S(v) = q) \\ \mathtt{sawRS}' = \mathtt{sawRS} \vee \\ (\exists v \in SCC_e)(\exists p \in \mathbb{P} \setminus \{\pi\})\ \mathsf{proc}_S(v) = p \wedge \mathtt{lastisRec}(p) \wedge p \in P \cap Q \end{array}}{(P, Q, \mathtt{cnt}, \mathtt{sawRS}, \mathtt{lastisRec}) \xRightarrow[\mathsf{bad}]{e,k} (P', Q', \mathtt{cnt}', \mathtt{sawRS}', \mathtt{lastisRec}')}$$

Fig. 7: Definition of the relation $\xRightarrow[\mathsf{bad}]{e,k}$

*and at least one of the two holds: either* $\mathtt{sawRS} = \mathsf{True}$*, or* $\mathtt{cnt} = k + 2$*.*

*Comparison with [4].* As for the notion of feasibility, to determine if an execution is bad, in [4] the authors use a monitor that builds a path between the send to process $\pi$ and the send from $\pi$. In addition to the problems related to the wrong characterisation of $k$-synchronizability, this monitor not only can detect an $RS$ edge when there should be none, but also it can miss them when they exist. In general, the problem arises because the path is constructed by considering only an endpoint at the time.

We can finally conclude that:

**Theorem 4.** *The $k$-synchronizability of a system $\mathfrak{S}$ is decidable for $k \geq 1$.*

## 6 *k*-synchronizability for Peer-to-Peer Systems

In this section, we will apply $k$-synchronizability to peer-to-peer systems. A peer-to-peer system is a composition of communicating automata where each pair of machines exchange messages via two private FIFO buffers, one per direction of communication. Here we only give an insight on what changes with respect to the mailbox setting.

Causal delivery reveals the order imposed by FIFO buffers. Definition 4 must then be adapted to account for peer-to-peer communication. For instance, two messages that are sent to a same process $p$ by two different processes can be received by $p$ in any order, regardless of any causal dependency between the two sends. Thus, checking causal delivery in peer-to-peer systems is easier than in the mailbox setting, as we do not have to carry information on causal dependencies.

Within a peer-to-peer architecture, MSCs and conflict graphs are defined as within a mailbox communication. Indeed, they represents dependencies over machines, i.e., the order in which the actions can be done on a given machine, and over the send and the reception of a same message, and they do not depend on the type of communication. The notion of $k$-exchange remains also unchanged.

**Decidability of Reachability for $k$-synchronizable Peer-to-Peer Systems.** To establish the decidability of reachability for $k$-synchronizable peer-to-peer systems, we define a transition relation $\xRightarrow[\mathsf{cd}]{e,k}{}^{\mathsf{p2p}}$ for a sequence of action $e$ describing a $k$-exchange. As for mailbox systems, if a send action is unmatched in the current $k$-exchange, it will stay orphan forever. Moreover, after a process $p$ sent an orphan message to a process $q$, $p$ is forbidden to send any matched message to $q$. Nonetheless, as a consequence of the simpler definition of causal delivery, , we no longer need to work on the conflict graph. Summary nodes and extended edges are not needed and all the necessary information is in function $B$ that solely contains all the forbidden senders for process $p$.

The characterisation of a $k$-synchronizable execution is the same as for mailbox systems as the type of communication is not relevant. We can thus conclude, as within mailbox communication, that reachability is decidable.

**Theorem 5.** *Let $\mathfrak{S}$ be a $k$-synchronizable system and $\vec{l}$ a global control state of $\mathfrak{S}$. The problem whether there exists $e \in asEx(\mathfrak{S})$ and* $\mathtt{Buf}$ *such that $(\vec{l_0}, \mathtt{Buf}_0) \overset{e}{\Rightarrow} (\vec{l}, \mathtt{Buf})$ is decidable.*

**Decidability of $k$-synchronizability for Peer-to-Peer Systems.** As in mailbox system, the detection of a borderline execution determines whether a system is $k$-synchronizable.

The relation transition $\xRightarrow[\mathsf{feas}]{e,k}{}^{\mathsf{p2p}}$ allows to obtain feasible executions. Differently from the mailbox setting, we need to save not only the recipient $\mathtt{dest}_\pi$ but also the sender of the delayed message (information stored in variable $\mathtt{exp}_\pi$). The transition rule then checks that there is no message that is violating causal delivery, i.e., there is no message sent by $\mathtt{exp}_\pi$ to $\mathtt{dest}_\pi$ after the deviation. Finally the recognition of bad execution, works in the same way as for mailbox systems. The characterisation of a bad execution and the definition of $\xRightarrow[\mathsf{bad}]{e,k}{}^{\mathsf{p2p}}$ are, therefore, the same.

As for mailbox systems, we can, thus, conclude that for a given $k$, $k$-synchronizability is decidable.

**Theorem 6.** *The $k$-synchronizability of a system $\mathfrak{S}$ is decidable for $k \geq 1$.*

## 7 Concluding Remarks and Related works

In this paper we have studied $k$-synchronizability for mailbox and peer-to-peer systems. We have corrected the reachability and decidability proofs given in [4]. The flaws in [4] concern fundamental points and we had to propose a considerably different approach. The extended edges of the conflict graph, and the graph-theoretic characterisation of causal delivery as well as summary nodes, have no equivalent in [4]. Transition relations $\xRightarrow[\mathsf{feas}]{e,k}$ and $\xRightarrow[\mathsf{bad}]{e,k}$ building on the graph-theoretic characterisations of causal delivery and $k$-synchronizability, depart considerably from the proposal in [4].

We conclude by commenting on some other related works. The idea of "communication layers" is present in the early works of Elrad and Francez [8] or Chou and Gafni [7]. More recently, Chaouch-Saad *et al.* [6] verified some consensus algorithms using the Heard-Of Model that proceeds by "communication-closed rounds". The concept that an asynchronous system may have an "equivalent" synchronous counterpart has also been widely studied. Lipton's reduction [14] reschedules an execution so as to move the receive actions as close as possible from their corresponding send. Reduction recently received an increasing interest for verification purpose, e.g. by Kragl *et al.* [12], or Gleissenthal *et al.* [11].

Existentially bounded communication systems have been studied by Genest *et al.* [10,15]: a system is existentially $k$-bounded if any execution can be rescheduled in order to become $k$-bounded. This approach targets a broader class of systems than $k$-synchronizability, because it does not require that the execution can be chopped in communication-closed rounds. In the perspective of the current work, an interesting result is the decidability of existential $k$-boundedness for deadlock-free systems of communicating machines with peer-to-peer channels. Despite the more general definition, these older results are incomparable with the present ones, that deal with systems communicating with mailboxes, and not peer-to-peer channels.

Basu and Bultan studied a notion they also called synchronizability, but it differs from the notion studied in the present work; synchronizability and $k$-synchronizability define incomparable classes of communicating systems. The proofs of the decidability of synchronizability [3,2] were shown to have flaws by Finkel and Lozes [9]. A question left open in their paper is whether synchronizability is decidable for mailbox communications, as originally claimed by Basu and Bultan. Akroun and Salaün defined also a property they called stability [1] and that shares many similarities with the synchronizability notion in [2].

Context-bounded model-checking is yet another approach for the automatic verification of concurrent systems. La Torre *et al.* studied systems of communicating machines extended with a calling stack, and showed that under some conditions on the interplay between stack actions and communications, context-bounded reachability was decidable [13]. A context-switch is found in an execution each time two consecutive actions are performed by a different participant. Thus, while $k$-synchronizability limits the number of consecutive sendings, bounded context-switch analysis limits the number of times two consecutive actions are performed by two different processes.

As for future work, it would be interesting to explore how both context-boundedness and communication-closed rounds could be composed. Moreover refinements of the definition of $k$-synchronizability can also be considered. For instance, we conjecture that the current development can be greatly simplified if we forbid linearisation that do not correspond to actual executions.
